# Case report: Significant response of alpha-fetoprotein-producing gastric cancer from combined chemotherapy and immunotherapy

**DOI:** 10.3389/fimmu.2024.1448875

**Published:** 2024-10-28

**Authors:** Xue Da, Zhang Juan, Hu Zhijun, Lyu Zhongchuan

**Affiliations:** ^1^ Department of General Surgery, Yantai Yuhuangding Hospital, Shandong, China; ^2^ Department of Pharmacy, Yantai Yuhuangding Hospital, Shandong, China

**Keywords:** case report, alpha-fetoprotein-producing gastric cancer (AFPGC), tislelizumab, chemotherapy, Immunotherapy, Alpha-fetoprotein (AFP)

## Abstract

**Background:**

Alpha-fetoprotein-producing gastric cancer (AFPGC) represents a particularly aggressive subtype of gastric carcinoma characterized by elevated rates of vascular invasion, lymphatic dissemination, hepatic metastasis, and an unfavorable clinical outcome. Treatment strategies for AFPGC have historically lacked specificity. Herein, a case is presented involving AFPGC in which the patient exhibited a notable response to combined anti-PD-1 antibody immunotherapy and SOX chemotherapy, potentially achieving a cure. This report marks the first application of this regimen in neoadjuvant therapy for AFP gastric cancer, followed by radical resection and postoperative adjuvant therapy.

**Case summary:**

A 62-year-old male patient presented with persistent upper abdominal distension and discomfort lasting over 2 months. Initial investigations revealed markedly elevated serum alpha-fetoprotein (AFP) levels, and subsequent pathological examination confirmed the diagnosis of AFPGC via gastroscopy. Due to the patient’s condition, surgical resection was initially deemed unfeasible. Therefore, a chemo-immunotherapy regimen consisting of SOX chemotherapy and the PD-1 inhibitor tislelizumab was administered for 3 cycles. Following this, successful laparoscopic radical gastrectomy was performed. The treatment protocol was continued with an additional 3 cycles postoperatively. At the time of this case report, the patient maintained a good quality of life with no evidence of disease recurrence or adverse events.

**Conclusion:**

The present report highlights a case of AFPGC where significant therapeutic success was achieved through a combined regimen of chemotherapy and immunotherapy, both before and after surgery. The use of anti-PD-1 antibody (tislelizumab) in combination with SOX regimen (S-1 and oxaliplatin) demonstrated effective treatment of AFPGC, potentially offering a curative approach. This approach represents a promising targeted therapy option for patients with AFPGC.

## Introduction

As a common malignant tumor, gastric cancer poses a serious threat to human health due to highly mortality and low early diagnosis rate. Alpha-fetoprotein producing gastric cancer (AFPGC) is a distinct subtype of gastric cancer characterized by lower incidence but higher rates of recurrence and metastasis. These tendencies are thought to be influenced by the specific biological properties associated with the AFP molecule (1). At present, treatment strategies for AFPGC primarily rely on conventional approaches used for gastric cancer, lacking specific targeted therapies (2). Preoperative neoadjuvant therapy has shown promise in enhancing the curative potential of AFPGC. However, SOX chemotherapy alone is associated with a low remission rate and significant drug resistance (3), contributing to high rates of tumor recurrence. The treatment approach for AFPGC should not be uniformly applied as for conventional gastric cancer. Currently, there are no standardized guidelines for AFPGC treatment, and strategies often rely on individual clinical experience. Various therapeutic approaches are still under active investigation and exploration to optimize outcomes for AFPGC patients.

As a critical part of tumor treatment, immunotherapy aims to mobilize the body’s immune system to target tumor cells more specifically, leading to their recognition, engulfment, and destruction by the body’s immune defenses. The PD-L pathway is a significant pathway of immunotherapy, where specific molecules on tumor surfaces exploit this pathway to evade immune detection. Blocking PD-1 (L1) can enhance tumor aggressiveness, leading to increased recurrence and metastasis. Tislelizumab, approved by the National Medical Products Administration of China and the US Food and Drug Administration (FDA) for hepatocellular carcinoma (HCC) (4, 5). The SOX regimen, widely used in gastric cancer treatment, has shown limited efficacy in AFPGC. Herein, a case is presented involving AFPGC where, for the first time, a patient received tislelizumab combined with the SOX regimen for 3 cycles prior to successful tumor removal. Subsequently, the same regimen was continued for 3 additional cycles postoperatively, and as of 15 months follow-up, there has been no disease progression.

## Case report

**Figure 1:**
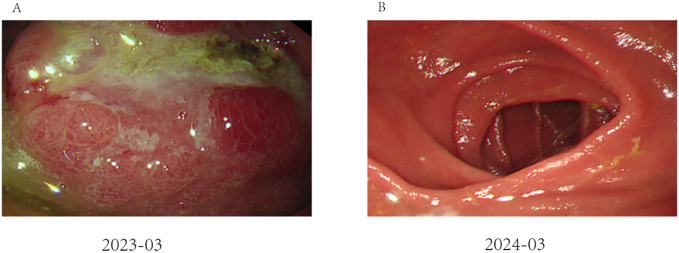
Endoscopic performance before and after treatment: **(A)** Image of gastroscopy results, showing a tumor located at the antrum of the stomach (as indicated by the arrows); **(B)** The remnant stomach and anastomosis were unobstructed, and there was no tumor recurrence.

**Figure 2:**
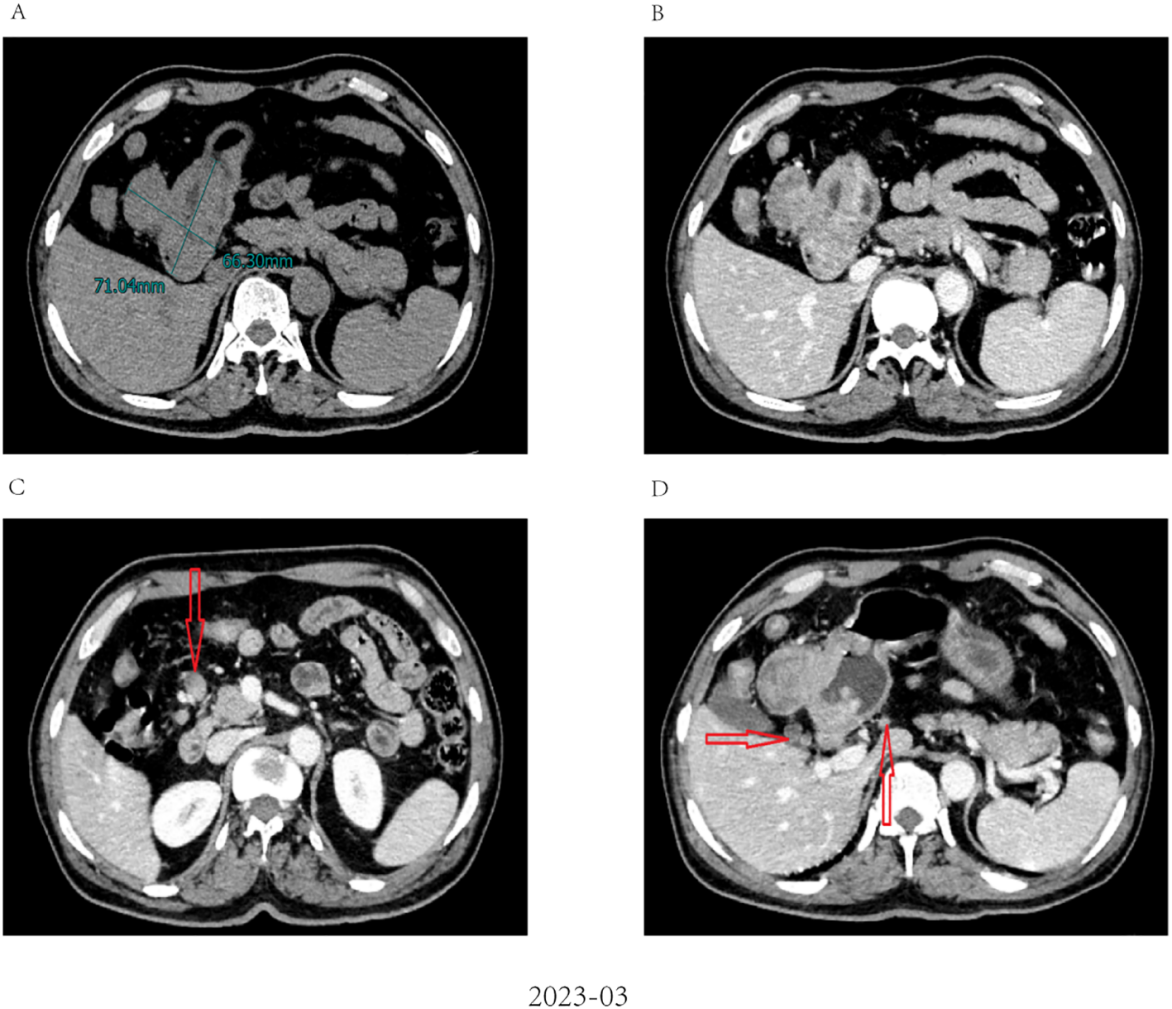
Abdominal CT imaging before chemo-immunotherapy **(A)**. Contrast-enhanced CT revealed a large tumor measuring 71 × 66 mm in size; **(B)** The wall of the gastric antrum was thickened and enhanced; **(C, D)** Contrast-enhanced CT revealed multiple enlarged abdominal lymph nodes (red arrow).

A 62-year-old male patient was admitted to our hospital in March 2023 due to discomfort in the upper abdomen for more than 2 months, accompanied by nausea, acid reflux, and occasional vomiting. The gastroscopy conducted at our hospital revealed a large tumor occupying a significant portion of the antrum. Pathological examination identified it as a poorly differentiated adenocarcinoma (**Figure 1A**). Immunohistochemical analysis indicated positivity for CK8 and CK18, with a Ki-67 proliferation index of 15%, and showed c-erbB-2(-). Microsatellite stability testing showed the positive expression of mismatch repair proteins MLH1, MSH6, PMS2, and MSH2, classifying the tumor as microsatellite-stable (MSS) type. PD-L1 expression level was also tested using the PD-L1.IHC 22C3 pharm Dx, revealing a negative PDL1 expression. Computed Tomography (CT) imaging revealed a large tumor measuring 71 × 66 mm in size, with thickening and enhancement of the gastric antrum wall. Multiple enlarged lymph nodes were observed, suggestive of metastasis (**Figure 2**). Tumor marker AFP levels were markedly elevated at 24233 ng/L (normal level: < 12 ng/ml), following exclusion of hepatitis, liver cirrhosis, and other conditions associated with AFP elevation. As such, the patient was diagnosed as having locally advanced AFPGC.

**Figure 3:**
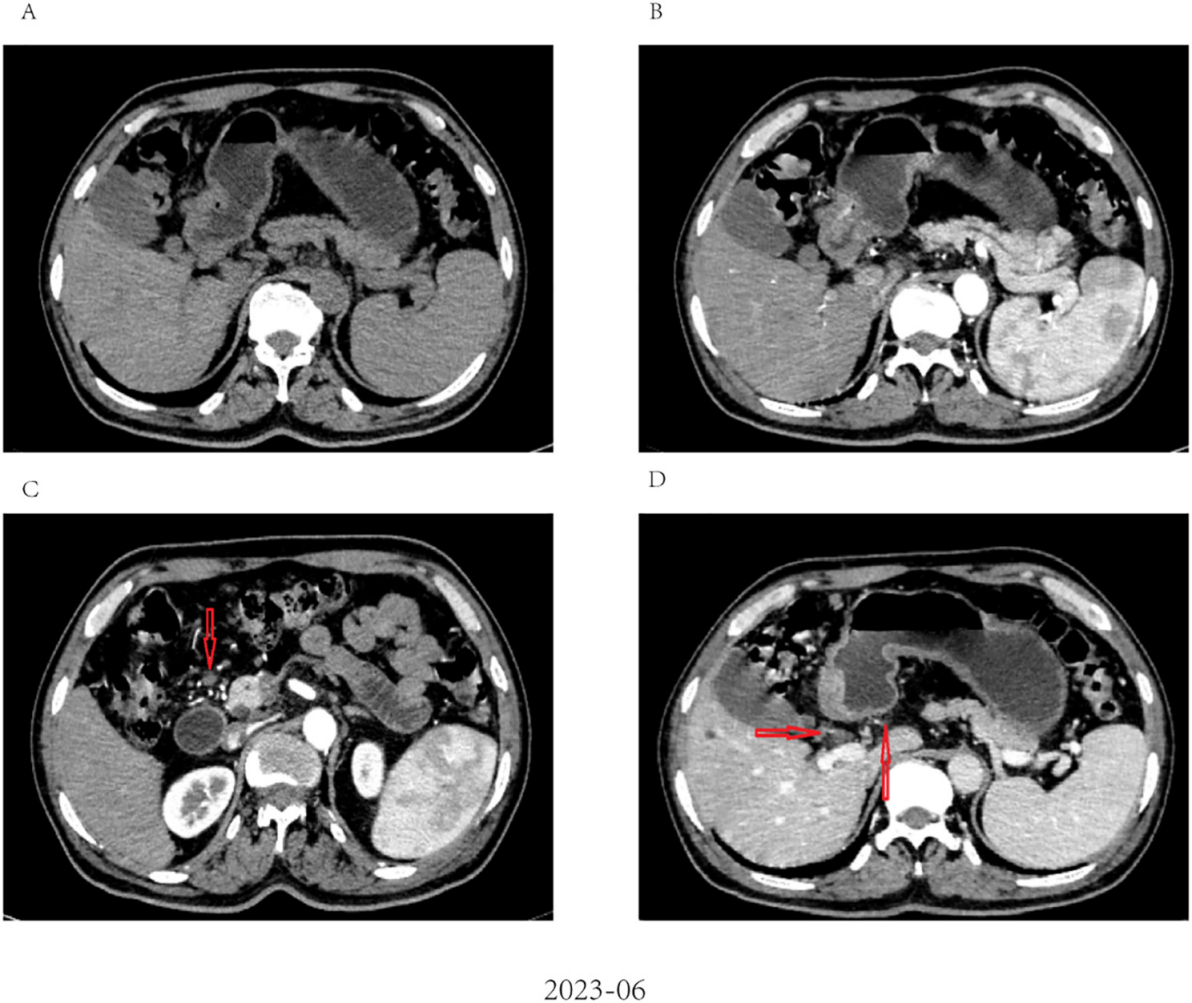
Radiological response evaluation of stomach and abdominal lymph nodes during the clinical course. **(A, B)** partial response (PR) was confirmed by contrast-enhanced CT, which showed a dramatic reduction in size of tumor after three cycles of tislelizumab combined with SOX; **(C, D)** Contrast-enhanced CT showed that the size of the abdominal lymph nodes was reduced after three cycles of tislelizumab combined with SOX (red arrow).

After evaluating the patient, surgical resection was deemed unfeasible, leading to a multidisciplinary decision to initiate neoadjuvant therapy. The regimen included intravenous administration of the anti-PD-1 antibody tislelizumab (200mg) once every 21 days, combined with the SOX regimen. S-1 (40 mg/m2, bid) was taken orally from day 1 to 14, and oxaliplatin (130 mg/m2) was administered intravenously on day 1 of each cycle. After three cycles of this combined therapy, the size of the largest tumor decreased significantly to approximately 20 × 30 mm (**Figure 3**). Evaluation also showed a progressive decline in AFP levels from 24233 ng/L to 3.38 ng/L. Hematological retests conducted after each cycle (April, May, and June 2023) consistently demonstrated a marked reduction in AFP levels (**Table 1**). The treatment response was classified as partial response (PR). Throughout the treatment, the patient experienced manageable side effects such as fatigue and nausea, which were effectively managed with symptomatic therapies.

**Table 1:** Serum AFP level trends in patient during treatment and re-examination (ng/ml).

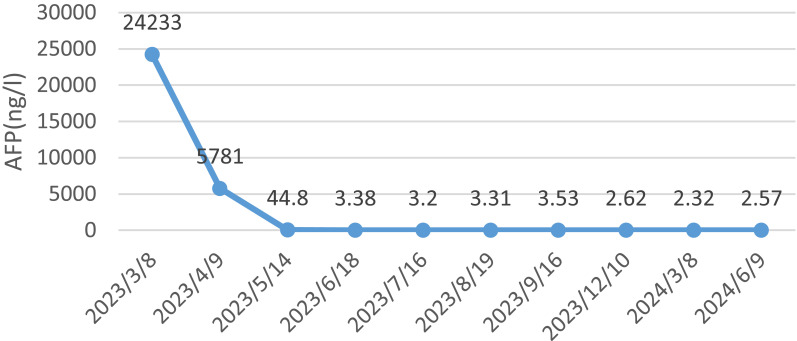

**Figure 4:**
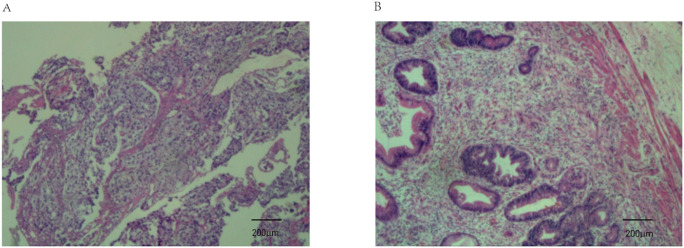
Pathological findings from endoscopic biopsy before chemo-immunotherapy and postoperative pathology. **(A)** Endoscopic biopsy before chemo-immunotherapy revealed poorly differentiated gastric adenocarcinoma (haematoxylin and eosin staining; magnification, ×100); **(B)** Postoperative pathology indicated tumor regression (AJCC/CAP).

A multidisciplinary team (MDT) evaluation showed that surgical resection was possible, and laparoscopic radical gastrectomy was successfully performed. Resected tissue analysis revealed a small number of residual tumor cells, with the majority showing signs of degeneration and cell death. Postoperative pathology indicated tumor regression of grade 1 according to AJCC/CAP criteria. Notably, no lymph node metastases were detected in any regional group, resulting in a TNM stage classification of T1aN0M0 (**Figure 4**).

**Figure 5:**
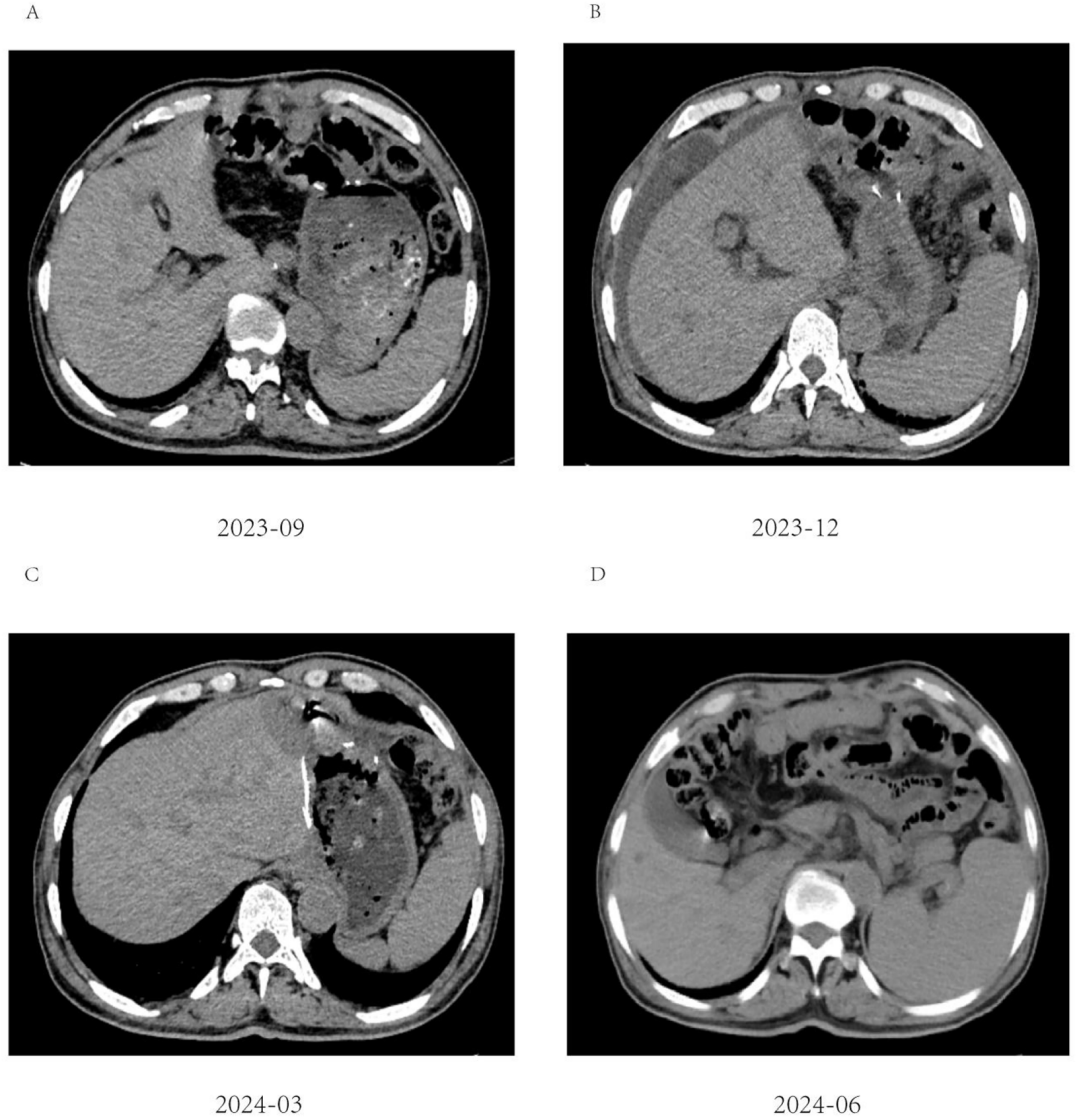
Abdominal CT imaging during follow-up. **(A-D)** Postoperative CT showed that the patient’s condition was stable, and no tumor recurrence was found; **(B)** Unexplained abdominal effusion was detected in 2023-12, with no accompanying discomfort. No special treatment was administered, and a review after 3 months showed that the peritoneal effusion had disappeared.

Numerous tumor markers, including AFP, were within the normal range. Considering the presence of residual tumor cells in the specimens, three cycles of postoperative adjuvant therapy were administered. Tumor markers were re-evaluated in July, August, and September 2023, and abdominal CT scans were performed in September 2023. Following this, all treatments were discontinued, and follow-up was initiated. Tumor markers were assessed in December 2023, February 2024, and May 2024. Abdominal CT scans performed in September and December 2023, as well as in March and June 2024, showed no disease progression (**Figure 5**).

**Figure 6:**
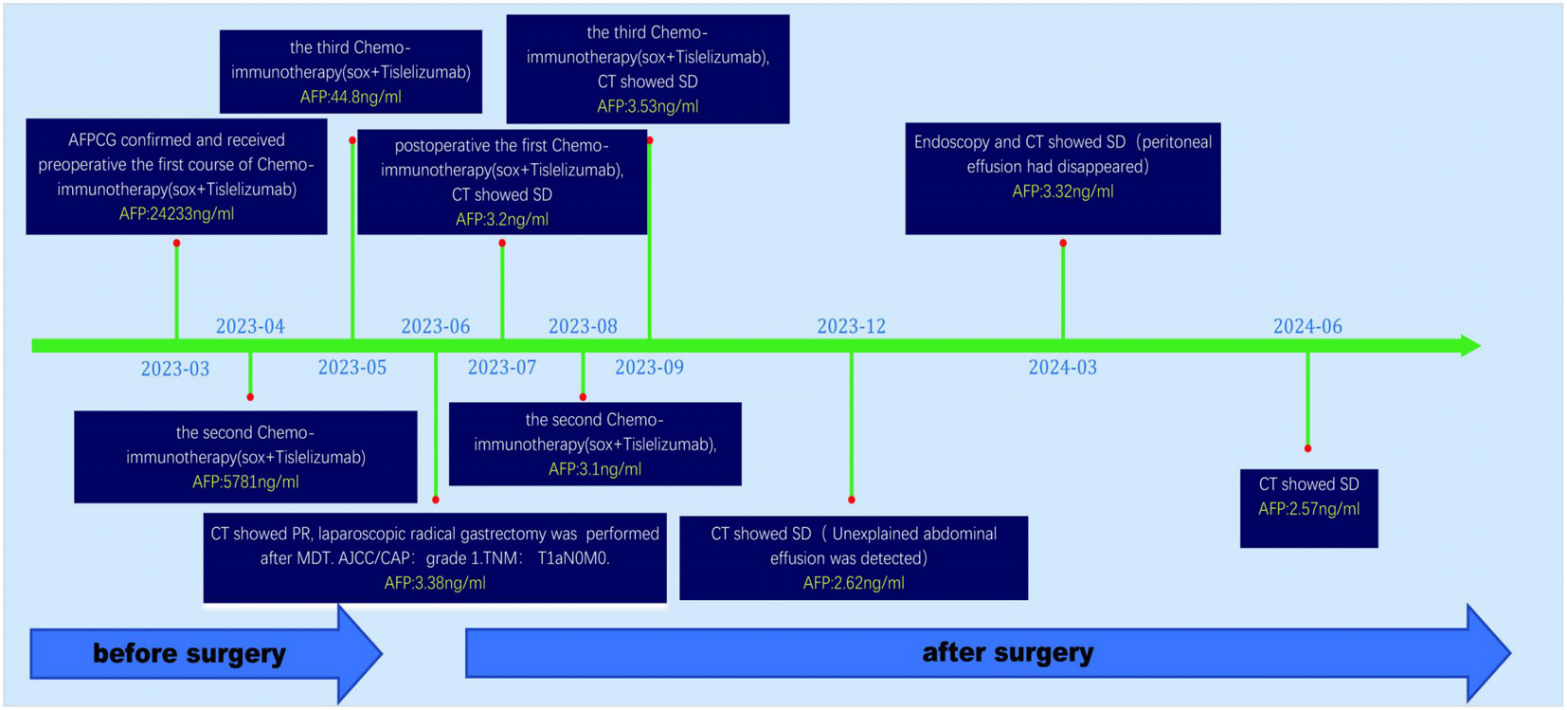
Timeline of the clinical course. PR, Partial Response; SD, Stable Disease.

Follow-up gastroscopies were also performed (**Figure 1**). At the time this case report was written, 15 months had passed, and the patient has had a good quality of life without any signs of recurrence or adverse events. The patient’s treatment timeline is shown in **Figure 6**.

## Discussion

Alpha fetoprotein (AFP) was first discovered in human serum by Bergstrandh et al. (6) and is one of the commonly used tumor markers in clinical practice. It is synthesized mainly in liver tissue and yolk sac (7). AFP can inhibit T lymphocyte mediated cytotoxicity, change the proportion of T lymphocytes such as CD4+ and CD8+, and inhibit the immune function of the human body (8). AFP may also evade immune surveillance by regulating tumor necrosis factor receptor, c-Jun and Fas pathways (9). Further, AFP inhibits the maturation of dendritic cells *in vivo*, which play a key role in immunity. As a significant oncology marker of primary liver cancer, AFP can be highly expressed in more than 60% of patients with primary liver cancer. However, abnormal high expression of AFP can also be detected in some patients with non-liver cancer solid tumous, such as rectal cancer, ovarian cancer and uterine tumor.

Ever since it was first identified as “AFP-producing gastric cancer” in the 1970s (6), AFPGC has remained under recognized due to its rarity (10). The AFP level in the serum of an AFPGC patient is higher than the normal reference value and can even exceed the limit of the monitoring range. It is generally believed that AFPGC can be diagnosed when the AFP level exceeds 20 ng/mL. Similar to gastric cancer, AFPGC is usually diagnosed histologically after endoscopic biopsy and staged using CT, endoscopic ultrasound, PET, and laparoscopy.

Ishikura et al. proposed the term “hepatoid adenocarcinoma of the stomach, HAS” for a gastric cancer with the histological features of hepatocytic differentiation (11), Which is the another subtype of gastric cancer closely related to APF. Therefore, AFPGC and HAS have overlapping but distinct populations. The former is more concerned with serum AFP level, while the latter is mainly focusing on pathological morphology.

The marked clinical features of AFPGC are high invasiveness, early metastasis, and rapid progress. Research by Liu et al. of 104 patients of 104 patients found that the incidence of liver metastasis was 60.6%, with a median time of 7.4 months from the detection of liver metastasis to surgery, which is far shorter than that (20.6 months) of common gastric adenocarcinoma (12). Zhang et al. showed 13.9 months of median OS in 105 patients with advanced AFPGC, which is far shorter than that (30.6months) of common gastric cancer (13).

In another study of postoperative survival of AFPGC, Takayo Ota et al. searched the literature in PubMed up to 30 September 2022 they identified 23 studies of AFPGC (14). The median overall survival with AFPGC after curative surgery is 29–72 months, and the 5-year survival rate is 25.0–66.0%. Although the survival period and survival rate vary from study to study, the survival is longer for other GCs than for AFPGC in each study (15–17).

Tumor immunotherapy has been a significant research focus over the past 20 years. The concept that deficiencies in both active and passive immune responses contribute to tumor progression has gained increasing acceptance among scholars. Recent years have seen significant research progress in tumor immunotherapy across various solid tumors such as gastric cancer, colorectal cancer, liver cancer, and ovarian cancer (18).

PD-1, as the most widely studied immune checkpoint molecule, was discovered in 1992 as an apoptosis-related gene target (19). PD-1/PD-L1 antibodies targeting the PD-1/PD-L1 axis can effectively block this pathway and restore anti-tumor immune response (20, 21).

Previous studies demonstrated that chemotherapy might be able to enhance anticancer immunity by reactivating immune effector cells, stimulating tumor antigen presentation, and eliminating immune suppressor cells, thus resulting in a synergistic anticancer effect compared with the anti-PD-1monotherapy (22). this phenomenon has also been reported small cell lung cancer (23), leiomyosarcoma (24), non-small cell lung cancer (21), and bladder carcinoma (25).However, studies specific to AFPGC remain limited, and its mechanisms have not been fully elucidated.

Immunotherapy has shown some effect in the treatment of advanced gastric cancer. ATTRACTION-2 and KEYNOTE-059 studies have proved the efficacy of PD-1 mAb in the third-line treatment of gastric cancer (26, 27). However, the effectiveness of PD-1 antibody in the first-line treatment of gastric cancer is still controversial. The Phase III RCT clinical study KEYNOTE-062 showed that PD-1 antibody combined with chemotherapy was not inferior to chemotherapy in the first-line treatment of gastric cancer (28). The results of the Check Mate 649 study reported in ESMO 2020 indicated that PD-1 antibody had a survival advantage over chemotherapy alone (PFS 7.7 vs 6.9 months, OS 13.8 vs 11.6 months) (29). However, the improvement was still not ideal. In a real-world study, Lu conducted a real-world study. Which demonstrated that the PD-1 antibody Nivarumab, in combination with chemotherapy, provided substantial benefits to gastric cancer patients, showing significantly higher objective response rates (85.7% vs. 21.4%, P<0.005) and longer median progression-free survival (22.0 months vs. 5.0 months, P<0.001) compared to the control group (30). Nevertheless, to our knowledge, there are no published data on the effectiveness of immunotherapy for AFPGC.

Moreover, anti-PD-1 antibodies have also been approved for treating patients with advanced HCC. As a prominent tumor marker in liver cancer, elevated AFP levels are common, suggesting potential overlapping pathogenic mechanisms with AFPGC. Sorafenib, a tyrosine kinase inhibitor approved as a first-line treatment for HCC, has shown efficacy in AFPGC (31). AFP has thus could been explored as a therapeutic target for AFPGC (20). Studies have demonstrated that tumor cell apoptosis is accelerated when the AFP gene is knocked out (20). Luo P et al. found that the efficacy of relevant targeted drugs for AFP was positively correlated with the expression of AFP in tumor cells (32).

Meanwhile, the decline in the serum AFP level were significantly associated with PFS which were independent factors The study from Wang R showed that the PFS were significantly higher in patients whose serum AFP level decreased by ≥50% than in the patients whose AFP decreased by <50% (90.6% vs 51.5%, 46.8% vs 9.7% and 13.8% vs 4.9%, respectively, all P<.05) (33).

As such, if the mechanism of PD-1 treatment for liver cancer involves inhibition of AFP, it could potentially be applicable to AFPGC. This suggests a potential association between AFPGC and HCC based on AFP involvement.

While the Sox chemotherapy regimen is widely used in gastric cancer, its effectiveness in AFPGC is limited. Chemotherapy may enhance anticancer immunity by reactivating immune effector cells, promoting tumor antigen presentation, and eliminating immune suppressor cells. This synergistic effect can lead to better outcomes compared to anti-PD-1 monotherapy (22, 34). In the present study, given the limited options for chemotherapy drugs in AFPGC, the Sox regimen could not be disregarded (35).

Deng’s study first reported a hepatoid adenocarcinoma of the stomach (HAS) patient achieving a pathologic complete response (PCR) with combined chemotherapy and immunotherapy, despite negative PD-L1 expression (36). Although complete remission was not achieved in postoperative pathology, the neoadjuvant therapy demonstrated significant efficacy. The innovative postoperative treatment included three additional courses, well tolerated by patients with expectations of achieving complete remission, pending further confirmation. Currently, no similar reports have been identified.

In conclusion, the present report represents the first application of neoadjuvant chemotherapy (SOX) combined with immunotherapy (tislelizumab) in advanced gastric cancer with elevated AFP levels. And, for the first time, a patient received tislelizumab combined with the SOX regimen for 3 cycles prior to successful tumor removal. Subsequently, the same regimen was continued for 3 additional cycles postoperatively, and as of 15 months follow-up, there has been no disease progression. The molecular feasibility of this regimen targeting AFP was also explored.

However, due to the retrospective nature of this single-case study, a major limitation is the lack of sufficient evidence to conclusively support the benefits of combined treatment. Nonetheless, despite these challenges, encouraging results have been achieved, suggesting potential applications and new treatment directions for patients with advanced gastric cancer with elevated AFP levels. The present report opens up new avenues for further exploration in AFPGC treatment strategies.

## Data Availability

The raw data supporting the conclusions of this article will be made available by the authors, without undue reservation.
